# Lyso-DGTS Lipid Derivatives Enhance PON1 Activities and Prevent Oxidation of LDL: A Structure–Activity Relationship Study

**DOI:** 10.3390/antiox11102058

**Published:** 2022-10-19

**Authors:** Ali Khattib, Sanaa Musa, Majdi Halabi, Tony Hayek, Soliman Khatib

**Affiliations:** 1 Natural Compounds and Analytical Chemistry Laboratory, MIGAL—Galilee Research Institute, P.O. Box 831, Kiryat Shemona 11016, Israel; 2 The Rappaport Family Institute for Research in the Medical Sciences, Rambam Medical Center, Haifa 31096, Israel; 3 Department of Biotechnology, Tel-Hai College, Upper Galilee 12210, Israel; 4 Ziv Medical Center, Safed 13100, Israel

**Keywords:** lyso-DGTS, paraoxonase 1, docking, structure-activity relationship, antioxidant activities, LDL oxidation

## Abstract

Paraoxonase 1 (PON1) plays a role in regulating reverse cholesterol transport and has antioxidative, anti-inflammatory, antiapoptotic, vasodilative, and antithrombotic activities. Scientists are currently focused on the modulation of PON1 expression using different pharmacological, nutritional, and lifestyle approaches. We previously isolated a novel active compound from *Nannochloropsis* microalgae—lyso-diacylglyceryltrimethylhomoserine (lyso-DGTS)—which increased PON1 activity, HDL-cholesterol efflux, and endothelial nitric oxide release. Here, to explore this important lipid moiety’s effect on PON1 activities, we examined the effect of synthesized lipid derivatives and endogenous analogs of lyso-DGTS on PON1 lactonase and arylesterase activities and LDL oxidation using structure–activity relationship (SAR) methods. Six lipids significantly elevated recombinant PON1 (rePON1) lactonase activity in a dose-dependent manner, and four lipids significantly increased rePON1 arylesterase activity. Using tryptophan fluorescence-quenching assay and a molecular docking method, lipid–PON1 interactions were characterized. An inverse correlation was obtained between the lactonase activity of PON1 and the docking energy of the lipid–PON1 complex. Furthermore, five of the lipids increased the LDL oxidation lag time and inhibited its propagation. Our findings suggest a beneficial effect of lyso-DGTS or lyso-DGTS derivatives through increased PON1 activity and prevention of LDL oxidation.

## 1. Introduction

Atherosclerosis is the accumulation of fatty acids/lipids and oxidized lipids in the innermost layer, the intima, of the arteries. Advanced atherosclerotic plaques can penetrate the arterial lumen, inhibit blood flow, and lead to tissue ischemia. Atherosclerotic cardiovascular disease (CVD) remains a leading cause of vascular disease worldwide [1].

Epidemiological studies have demonstrated that low-density lipoprotein (LDL) and high-density lipoprotein (HDL) are independent causes of CVD. Clinically, lipoprotein disorders, such as an increase in LDL-cholesterol or decrease in HDL-cholesterol levels, are common characteristics of CVD [2].

Many lines of evidence suggest that the oxidation of LDL plays an important role in the pathogenesis of atherosclerosis. Animal models have shown that the accumulation of oxidized LDL (ox-LDL) in the circulation can be a well-known risk marker of human CVD, caused by atherosclerosis [3]. HDL has a key role in atherosclerosis inhibition due to its antiatherogenic properties, such as reverse cholesterol transport, antioxidant, anti-inflammatory, antiapoptotic, vasodilatory and cytoprotective effects, and improvement of endothelial function [4,5]. HDL loses its beneficial properties in certain pathological situations, such as diabetes, acute infections, and coronary artery disease (CAD), termed “dysfunctional HDL”. Oxidative modifications of HDL proteins and lipids have been demonstrated to be one of the main causes of HDL dysfunction [6].

There is considerable evidence that the antioxidant activity of HDL is largely related to its associated antioxidant enzyme paraoxonase 1 (PON1). Epidemiological evidence has shown that low PON1 activity is associated with an increased risk of cardiovascular events [7,8]. PON1 has many activities—hydrolysis of organophosphate triesters, aryl esters, cyclic carbamates, glucuronides, estrogen esters, and thiolactones—while its “natural” substrates are assumed to be lactones [9,10]. The aromatic nature of amino acids in the active site of PON1 could explain why the enzyme prefers lipophilic substrates [11]. All three members of the PON family (PON1, PON2, and PON3) share this property of being lactonases.

PON1 can hydrolyze lipid peroxide in lipoproteins through its lipolactonase activity, thereby decreasing oxidative stress in serum lipoproteins and macrophages and reducing inflammation [12,13]. These important features can markedly affect the development of atherosclerotic plaques and cardiovascular events [14]. Experiments with PON1-knockout mice have indicated that the absence of PON1 leads to an increase in endothelial adhesion molecules and oxidative stress, confirming this enzyme’s role in preventing the onset of atherosclerosis [9,15].

A clinical study suggested that the serum antioxidant activity of PON1 (arylesterase activity) is an important factor in protecting from oxidative stress and lipid peroxidation in CAD. Thus, evaluating the effects of PON 1 on CAD patients may be promising for the treatment and prognosis of this disease [16].

PON1 enzymatic activities can be affected by its interaction with elements in its environment. For example, dietary lipids and lipid-peroxidation products can decrease PON1 activity and gene expression; following fish oil supplementation, polyunsaturated fatty acids significantly decreased postprandial serum PON1 activity, associated with an increase in susceptibility of the serum to lipid peroxidation [17]. The effect has been confirmed in humans [18]. Conversely, the consumption of pomegranate juice, which is rich in polyphenols and several antioxidants, resulted in higher PON1 activity [19,20]. Other antioxidants, such as flavonoids, quercetin, and glabridin, also protect PON1 from oxidation and increased its lactonase activity [21,22].

We hypothesize that natural agents with the potential to increase PON1 function can improve its atheroprotective effects and may reduce CVD risk. We previously isolated a promising agent from *Nannochloropsis* sp. microalgae, determined to be lyso-diacylglyceryltrimethylhomoserine (lyso-DGTS), which significantly increased recombinant PON1 (rePON1) and HDL-cholesterol efflux and enhanced endothelial nitric oxide release [23,24]. In our previous studies, lyso-DGTS was found to protect macrophages and LDL from oxidation and potentially improve HDL function by increasing the activity of PON1 [24]. Moreover, we showed that lyso-DGTS lipid interacts with HDL proteins and with the HDL core, improving HDL functions in vitro and in vivo [25].

In the present study, to characterize this important lipid moiety’s effect on PON1 activities, we investigated the effect of synthesized lipid derivatives and endogenous analogs of lyso-DGTS on rePON1 lactonase and arylesterase activities using structure–activity relationship (SAR) methods. In addition, the biological importance of these interactions was evaluated by examining the effect of these bioactive lipids on LDL oxidation.

## 2. Experimental Procedures

### 2.1. Materials

Lyso-DGTS was isolated from *Nannochloropsis* microalgae in our laboratory. Lyso-DGTS analogs palmitic acid, cis-5,8,11,14,17-eicosapentaenoic acid (EPA), oleic acid, oleoyl chloride, EDC-HCl, DMAP, and dihydrocoumarin were purchased from Sigma-Aldrich. EPA chloride was purchased from Cayman Chemical. RePON1, generated in *Escherichia coli* via direct evolution, was purchased from the Weizmann Institute of Science (Rehovot, Israel).

### 2.2. Isolation of Lyso-DGTS

Lyso-DGTS was isolated and purified as described previously [23]. *Nannochlorpsis* sp. was extracted using 70% ethanol-water solvent to obtain extract in 20% yield. Lyso-DGTS was purified using preparative chromatography methods to obtain the pure compound, as determined via HPLC (purity = 97%), in ~1% yield of the extract.

### 2.3. Synthesis of Lyso-DGTS Derivatives

#### 2.3.1. General Procedure for the Synthesis of (*S*)-1-Carboxy-3-hydroxy-*N,N,N*-trimethylpropan-1-aminium (*l*-Homoserine Betaine) (Scheme 1)

*l*-Homoserine (1 g, 8.39 mmol) was dissolved in MeOH (20 mL) and the solution was cooled to 0 °C under a nitrogen atmosphere. SOCl_2_ (0.7 mL, 10.07 mmol) was added slowly while stirring. The reaction was stirred for 24 h at room temperature and the solvent was evaporated to obtain (*S*)-methyl 2-amino-4-hydroxybutanoate.

**Scheme 1:**
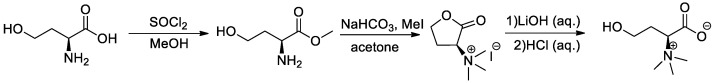
Synthesis of (*S*)-1-carboxy-3-hydroxy-*N,N,N*-trimethylpropan-1-aminium (*l*-homoserine betaine).

(*S*)-methyl 2-amino-4-hydroxybutanoate (1.118 g, 8.39 mmol) and NaHCO_3_ (5 g, 59.52 mmol) were suspended in 70 mL acetone. A solution of methyl iodide (3.13 mL, 50.42 mmol) in 3 mL acetone was slowly added to the suspension. The reaction mixture was stirred overnight at room temperature under a nitrogen atmosphere. The solution was filtered, and the filtrate was evaporated to dryness and used in the next step without further purification.

*N,N,N*-trimethyl-*l*-homoserine lactone (8.39 mmol) was dissolved in H_2_O (20 mL). LiOH (100 mg, 4.17 mmol) was added and the mixture was stirred for 30 min at room temperature. The reaction mixture was neutralized with 7% aqueous HCl, followed by evaporation. The residue was subjected to chromatography in a silica gel column eluted with (20%) methanol in dichloromethane to give (*S*)-1-carboxy-3-hydroxy-*N,N,N*-trimethylpropan-1-aminium (1.24 g, 95%) (*l*-homoserine betaine). 

#### 2.3.2. General Procedure for the Synthesis of *l*-Homoserine Betaine Fatty Acid Ester Derivatives (Scheme 2)

(*S*)-1-carboxy-3-hydroxy-*N,N,N*-trimethylpropan-1-aminium (1 equiv.) was dissolved in 2 mL anhydrous N,N-dimethylformamide (DMF) and cooled to 0 °C. N-(3-Dimethylaminopropyl)-*N*′-ethylcarbodiimide hydrochloride (EDC-HCl) (1.5 equiv.) and DMAP (1.2 equiv.) were added to the cooled solution. The mixture was stirred for 20 min under a nitrogen atmosphere; then, fatty acid derivative (1 equiv.) was added to the solution. The reaction mixture was stirred overnight at room temperature and then extracted with ethyl acetate. The organic layer was dried over sodium sulfate, filtered, and evaporated to dryness. The crude product was purified via chromatography on a silica gel column using dichloromethane: methanol as the eluent.

**Scheme 2:**
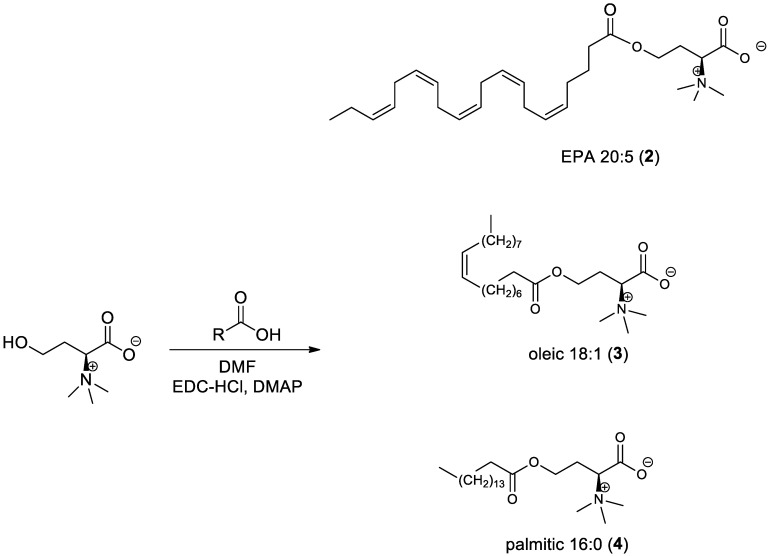
Synthesis of *l*-homoserine betaine fatty acid ester derivatives.

#### 2.3.3. General Procedure for the Synthesis of Amide-Fatty Acid Derivatives

##### (*S*)-2-((Tert-butoxycarbonyl) amino)-4-oleylamido butanoic acid (C2) and (*S*)-2-((Tert-butoxycarbonyl)amino)-4-((5Z,8Z,11Z,14Z,17Z)-eicosa-5,8,11,14,17-pentaenamido)butanoic acid (C3) (Scheme 3)

(*S*)-4-Amino-2-(tert-butoxycarbonylamino) butanoic acid (C1) (100 mg, 0.458 mmol) and *N,N*-diisopropylethylamine (290 mg, 2.243 mmol) were dissolved in 5 mL anhydrous DMF. Oleoyl chloride (135 mg, 0.448 mmol) was dissolved in anhydrous DMF (1 mL) and stirred at room temperature for 10 min under a nitrogen atmosphere. The mixture was added slowly to the solution of **C1** and the reaction mixture was stirred for 4 h at room temperature. DDW (20 mL) and chloroform (40 mL) were added to the mixture, and the aqueous layer was treated with 7% HCl solution until pH 3 was achieved. Then, it was extracted twice with chloroform (40 mL) and the organic layers were collected, dried over anhydrous Na_2_SO_4_, filtered, and evaporated. The residue was subjected to chromatography in a silica gel column eluted with 25% methanol in dichloromethane to give compound **C2**. Compound **C3** was prepared from eicosapentaenoyl chloride using a similar procedure.

**Scheme 3:**
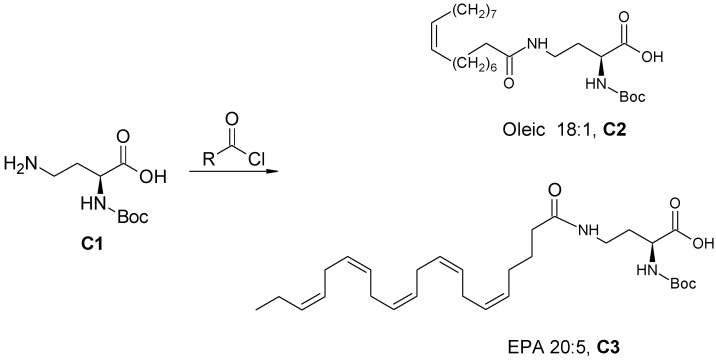
Synthesis of (*S*)-2-((tert-butoxycarbonyl) amino)-4-oleylamido butanoic acid (**C2**) and (*S*)-2-((tert-butoxycarbonyl)amino)-4-((5Z,8Z,11Z,14Z,17Z)-eicosa-5,8,11,14,17-pentaenamido)butanoic acid (**C3**).

##### (*S*)-2-Amino-4-oleylamido Butanoic Acid (C4) and (*S*)-2-Amino-4-((5Z,8Z,11Z,14Z,17Z)-eicosa-5,8,11,14,17-pentaenamido)butanoic Acid (C5) (Scheme 4)

(*S*)-2-((tert-butoxycarbonyl)amino)-4-((5Z,8Z,11Z,14Z,17Z)-eicosa-5,8,11,14,17-pentaenamido)butanoic acid (**C3**) was dissolved in DCM (10 mL). Trifluoroacetic acid (TFA; 2 mL, 26.09 mmol) was added and the reaction mixture was stirred at room temperature for 24 h to give **C5**, which was purified in a silica gel column eluted with 20% methanol in dichloromethane. Using a similar procedure, compound **C4** was prepared from **C2**.

**Scheme 4:**
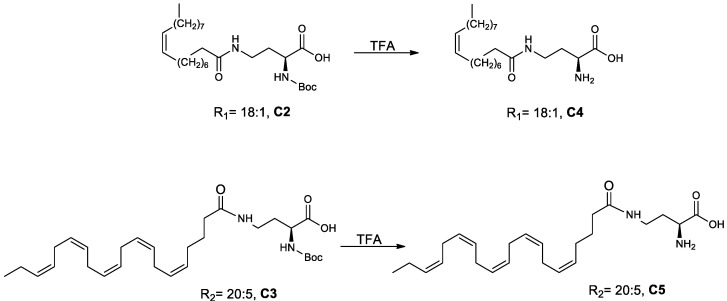
Synthesis of (*S*)-2-amino-4-oleylamido butanoic acid (**C4**) and (*S*)-2-amino-4-((5Z,8Z,11Z,14Z,17Z)-eicosa-5,8,11,14,17-pentaenamido)butanoic acid (**C5**).

##### 3-(*S*)-4-((5Z,8Z,11Z,14Z,17Z)-Eicosa-5,8,11,14,17-pentaenamido)-1-methoxy-*N,N,N*-trimethyl-1-oxobutan-2-aminium (5)) (Scheme 5)

(*S*)-2-amino-4-((5Z,8Z,11Z,14Z,17Z)-eicosa-5,8,11,14,17-pentaenamido)butanoic acid (**C5**) was dissolved in methanol (10 mL), and NaHCO₃ (2.19 g, 5.956 mmol) was added to the mixture while stirring. Iodomethane (MeI; 200 µL, 3.2 mmol) was dissolved in methanol (1 mL) and added to the reaction mixture. The reaction mixture was stirred at room temperature for 24 h and then evaporated. The crude product was subjected to chromatography in a silica gel column eluted with 35% methanol in dichloromethane to give compound **5**. Using a similar procedure, compound **6** was prepared from **C4**.

**Scheme 5:**
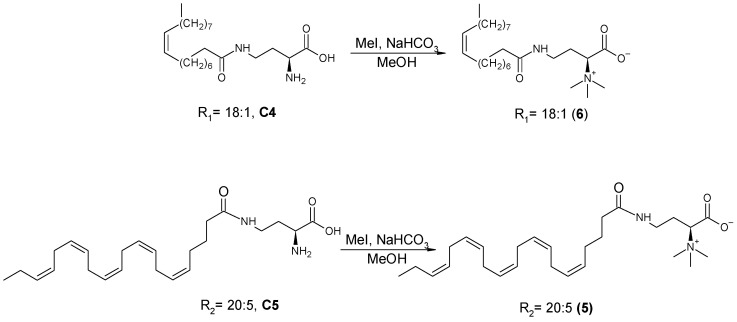
Synthesis of 3-(S)-4-((5Z,8Z,11Z,14Z,17Z)-eicosa-5,8,11,14,17-pentaenamido)-1-methoxy-N,N,N-trimethyl-1-oxobutan-2-aminium (**5**) and 1-carboxy-N-N-N-trimethyl-3-oleamidopropan-1-aminium (**6**).

### 2.4. Chromatography Analysis

#### 2.4.1. HPLC Analysis

HPLC analysis was performed with UHPLC connected to a photodiode array detector (Dionex Ultimate 3000), with a reverse-phase column (Phenomenex RP-18, 150 × 4.0 mm, 3 µm). The mobile phase was a mixture of (A) DDW with 0.1% formic acid and (B) acetonitrile with 0.1% formic acid with a flow of 1ml/min and gradient starting with 5% B and increasing in a concentration to 95% B for 30 min and then kept at 95% B for an additional 5 min.

#### 2.4.2. LC–MS Analysis

LC–MS analysis was performed with a Waters ZQ-mass spec system connected to an API single-quadrupole mass detector. ESI capillary voltage was set to 3800 V, capillary temperature to 250 °C, and gas flow to 60 mL/min.

### 2.5. NMR Analysis

The synthesized compounds were dissolved in deuterated methanol (CD_3_OD) or water (D_2_O), and ^1^H-NMR spectra were recorded at room temperature with a Bruker 400 MHz instrument with chemical shifts reported in ppm relative to the residual deuterated solvent.

#### 2.5.1. *l*-Homoserine Betaine

^1^H-NMR (400 MHz, CD_3_OD), δ: 3.69 (2H, m), 3.52 (1H, m), 3.13 (9H, s), 2.14 (1H, m), 1.99 (1H, m). (ESI+) *m/z*: 162. (NMR purity = 98%).

#### 2.5.2. Homoserine Betaine Eicosapentaenoate (**2**)

^1^H-NMR (400 MHz, CD_3_OD), δ: 5.45 (10H, m), 4.21 (1H, m), 4.14 (1H, m), 3.73 (1H, m), 3.29 (9H, s), 2.92 (8H, m), 2.08–2.51 (6H, m), 1.78 (2H, t), 1.29 (2H, m), 1.04 (3H, t). (ESI+) *m/z*: 446. HPLC Purity: 95%

#### 2.5.3. Homoserine Betaine Oleate (**3**)

^1^H-NMR (400 MHz, CD_3_OD), δ: 5.35 (2H, dt), 4.27 (1H, m), 4.13 (1H, m), 3.19 (1H, m), 3.21 (9H, s), 2.35 (2H, t), 2.04 (4H, m), 1.61 (2H, m), 1.30 (m, 22H), 0.90 (3H, t). (ESI+) *m/z*: 426. HPLC purity: 95%.

#### 2.5.4. Homoserine Betaine Palmitate (**4**)

^1^H-NMR (CD_3_OD, 400 MHZ), δ: 4.25 (1H, m), 4.12 (1H, m), 3.67 (1H, m), 3.19 (9H, s), 2.31 (2H, t), 2.22 (2H, m), 2.12 (2H, m) 1.61 (2H, q), 1.31 (25H, s), 0.90 (3H, t). (ESI+) *m/z*: 400. HPLC purity: 96%.

#### 2.5.5. EPA Amide-Lyso (**5**)

^1^H-NMR (600 MHz, CD_3_OD), δ: 1.01 (3H, t, J = 7 Hz), 1.72 (2H, q), 2.02(1H, m), 2.14 (4H, m), 2.22 (1H, m), 2.25 (2H, t, J = 7 Hz), 2.87 (8H, m), 3.25 (10H, s), 3.30 (1H, m), 3.37 (1H, m), 3.62 (1H, dd, J = 7 Hz), 5.40 (10H, m). (ESI+) *m/z*: 445. HPLC purity: >98%.

#### 2.5.6. Oleic Amide-Lyso (**6**)

^1^H-NMR (600 MHz, CD_3_OD), δ: 0.94 (3H, t, J = 7 Hz), 1.36 (21H, m), 1.65 (2H, m), 2.05 (5H, m), 2.23 (2H, t, J = 7 Hz), 2.27 (1H, m), 3.25 (9H, s), 3.30 (1H, m), 3.38 (1H, m), 3.61 (1H, dd, J = 7 Hz), 5.38 (2H, m). (ESI+) *m/z*: 425. HPLC purity: >98%.

### 2.6. Biological Assays

#### 2.6.1. Effect of the Lipids on rePON1 Lactonase and Arylesterase Activities

The synthesized and purchased lyso-DGTS derivatives and analogs (final concentration 10 and 20 µg/mL) were incubated with rePON1 for 2 and 4 h at 37 °C in Tris buffer pH 8.4 containing 1 mM CaCl_2_. These concentrations were chosen in accordance with our previous published studies which showed that, in these concentrations, lyso-DGTS has a positive effect on PON1 and HDL activities in vitro and in vivo without inducing a cytotoxic response [23,24,25].

Lactonase and arylesterase activities were performed using our previous procedure [24]. Briefly, a 10-µL aliquot of each solution was added to a 96-well UV microplate containing 90 µL Tris buffer and 1 mM CaCl_2_ in each well. Then, 100 µL of 2 mM dihydrocoumarin (for lactonase activity) or phenylacetate (for arylesterase activity) was added.

The substrate hydrolysis rate was measured at 270 nm every 15 s for 10 min using a Tecan Infinite 200 PRO plate reader. Nonenzymatic hydrolysis of the substrate was subtracted from the total rate of hydrolysis. One unit of activity was equal to the hydrolysis of 1 µmol dihydrocoumarin or phenylacetate per minute.

#### 2.6.2. Tryptophan (Trp) Fluorescence-Quenching Measurements

The assay was performed as previously published in our studies [25,26]. Briefly, in 200 μL Tris buffer in a black 96-well plate, 2 μL of bioactive lipid (in DMSO) at various final concentrations (1, 5, 10, 25, 50, 75, and 100 μg/mL) and rePON1 (final concentration 50 µg/mL) were incubated at 37 °C or 25 °C for 4 h; then, fluorescence measurements were performed in a plate reader (Tecan Infinite 200 PRO). The slit widths for both excitation and emission were set to 5 nm. Emission spectra were recorded from 300 to 450 nm with an excitation wavelength of 290 nm. The relative change in fluorescence (∆f) was calculated as (*Ix* − *Io*)/*Io*, where *Ix* and *Io* are the fluorescence intensities of rePON1 in the presence and absence of the bioactive lipid, respectively.

### 2.7. Molecular Docking

The crystal structure of PON1 (1V04) was retrieved from the Protein Data Bank (PDB). The enzyme was prepared for docking using the AutoDock Tools program, an accessory program that allows the user to interact with AutoDock 4.2 from a graphical user interface. Water molecules were removed from the PDB file. Polar hydrogen atoms were added and Kollman united atom charges were assigned. Ligand docking was carried out with the AutoDock 4.2 Lamarckian Genetic Algorithm (GA-LS).

### 2.8. Cu^2+^-Induced LDL Oxidation

Oxidation of LDL was carried out in a UV ELISA plate at 37 °C and measured using a Tecan Infinite 200 PRO plate reader. To each well 10 μL of rePON1 (0.005 mg/mL in PBS pH 7.4) incubated with the tested compounds (final concentration of 20 µg/mL in PBS pH 7.4 with 0.1% *v/v* DMSO) for 2 h at 37 °C or 2 μL of the tested compounds (final concentration of 20 µg/mL in PBS pH 7.4 with 0.1% DMSO) without rePON1 was added. Then, 6 μL LDL (final concentration of 0.05 mg protein/mL in PBS pH 7.4) was added to the reaction. Finally, 10 μL CuSO_4_ (10 µM in DDW) was added and PBS was added to a final volume of 100 µL. LDL oxidation was determined via continuous monitoring (every 5 min for 3 h) of conjugated diene formation at 234 nm. Lag time and rate constant (K) of the propagation phase were calculated via nonlinear regression using the Gompertz growth equation. Percent inhibition of LDL oxidation was calculated as: 100 − (K(LDL + sample)/K(LDL) × 100).

## 3. Results

### 3.1. Synthesis of Lyso-DGTS Derivatives

**Figure 1:**
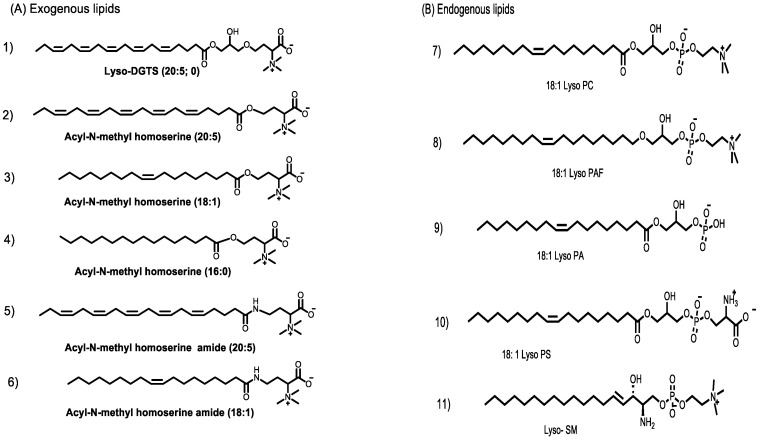
Synthesized derivatives of Lyso-DGTS (**A**) and lyso-DGTS analogs were purchased from (Sigma–Aldrich–Merck) (**B**). PC, phosphatidylcholine; PAF, platelet-activating factor; PA, phosphatidic acid; PS, phosphatidylserine; SM, sphingomyelin.

Five derivatives of lyso-DGTS were synthesized by binding different fatty acids—EPA, oleic acid, or palmitic acid—directly to the N-methylated homoserine via ester or amide bonds (compounds **2**–**6**; Figure 1A). In addition, six endogenous lipids which are analogs of lyso-DGTS were purchased (compounds **7**–**11**; Figure 1B). The effect of the lipids on the hydrolytic activities of the antioxidant enzyme PON1 and their ability to prevent LDL oxidation induced by copper ion were investigated, and SARs were determined to better understand the lipid moieties responsible for enhancing PON1 activities and preventing LDL oxidation.

### 3.2. Effect of Lyso-DGTS Derivatives and Endogenous Analogs on Lactonase Activity of rePON1

**Figure 2:**
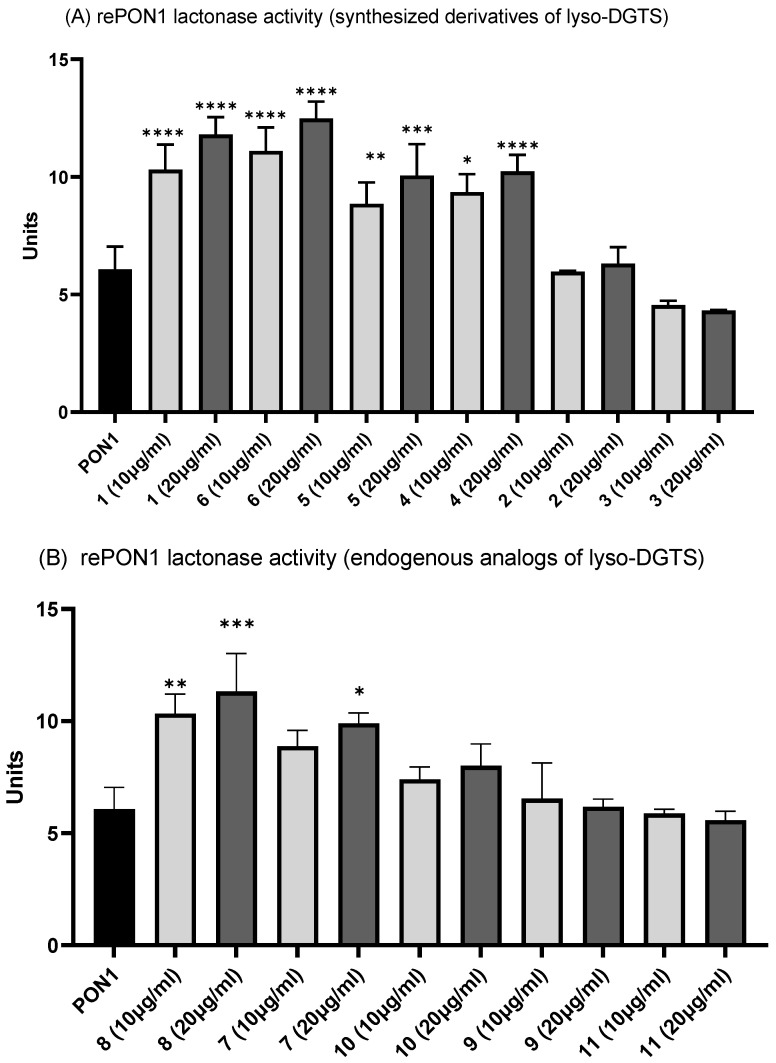
Lactonase activity of rePON1 after incubation with (**A**) synthesized derivatives of lyso-DGTS and (**B**) endogenous analogs of lyso-DGTS. RePON1 (5 µg/mL) was incubated with the lipid at concentrations of 10 and 20 µg/mL for 2 h at 37 °C in Tris buffer pH 8.4. Then, lactonase activity of rePON1 was measured via dihydrocoumarin assay and the results were expressed as units defined by the ability of rePON1 to hydrolyze 1 μM dihydrocoumarin in 1 min. Each experiment was repeated separately at least three times. * *p* ≤ 0.05, ** *p* ≤ 0.01, *** *p* ≤ 0.001, **** *p* ≤ 0.0001 relative to the control (PON1; rePON1 without bioactive lipid). Statistical analysis was via one-way ANOVA and Graphpad prism 9 software. See Figure 1 for compounds corresponding to each number.

The effect of the synthesized lyso-DGTS derivatives and analogs on rePON1 lactonase activity was examined and compared to that of natural lyso-DGTS isolated from *Nannochloropsis* microalgae. RePON1 lactonase activity was significantly increased, in a dose-dependent manner, when incubated with the natural lyso-DGTS for 2 h at 37 °C—from 5 units to 10 units and 12 units after incubation with lyso-DGTS at a concentration of 10 and 20 µg/mL, respectively. Similarly, incubation of rePON1 with the synthesized compounds **4**, **5**, and **6** at concentrations of 10 and 20 µg/mL increased its lactonase activity to 10 and 13 units, respectively. Compounds **2** and **3** did not affect the lactonase activity of rePON1—it was similar to rePON1 without lipids (Figure 2A). Incubation of rePON1 with the free fatty acids EPA, oleic acid, palmitic acid, and *l*-homoserine betaine also had no effect on its lactonase activity (data not shown).

The effect of the purchased endogenous lipids on rePON1 lactonase activity was also examined. Only two lipids—lyso-PAF (**8**) and lyso-PC (**7**)—increased the lactonase activity of rePON1 in a dose-dependent manner, from 5 units to 9 units and 13 units when the enzyme was incubated with the lipids at a concentration of 10 and 20 µg/mL, respectively. The other endogenous lipids—lyso-PS (**10**), lyso-PA (**9**), and lyso-SM (**11**)—did not affect rePON1 lactonase activity (Figure 2B).

### 3.3. Effect of Lyso-DGTS Derivatives and Endogenous Analogs on Arylesterase Activity of rePON1

**Figure 3:**
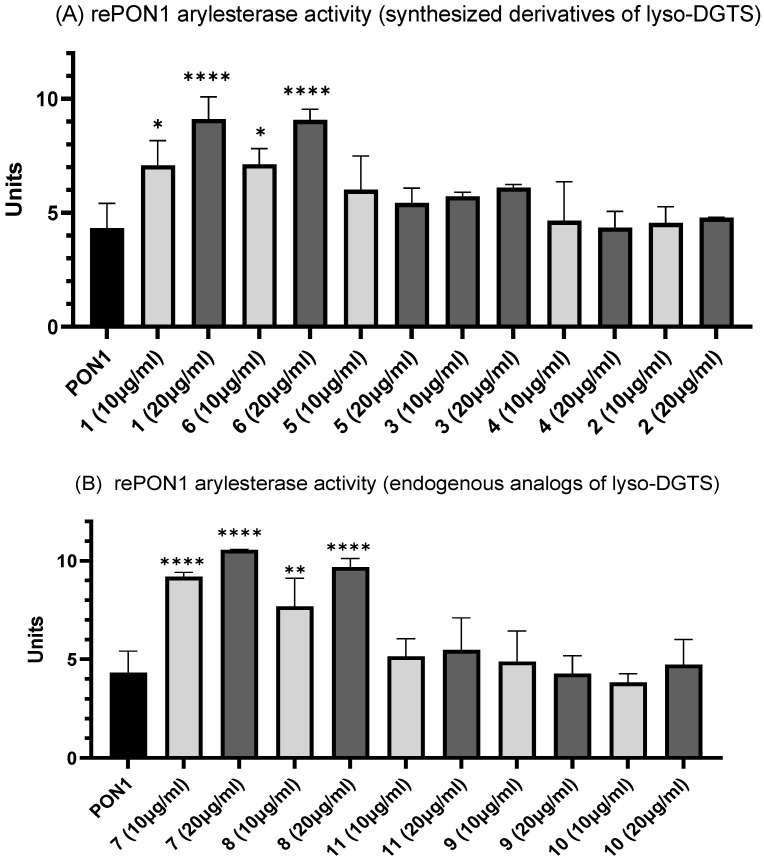
Arylesterase activity of rePON1 after incubation with (**A**) synthesized derivatives of lyso-DGTS and (**B**) endogenous analogs of lyso-DGTS. rePON1 (5 µg/mL) was incubated with the lipid at concentrations of 10 and 20 µg/mL for 2 h at 37 °C in Tris buffer pH 8.4. Then, arylesterase activity of rePON1 was measured via phenylacetate assay and the results were expressed as units defined by the ability of rePON1 to hydrolyze 1 mM phenylacetate in 1 min. Each experiment was repeated separately at least three times. * *p* ≤ 0.05, ** *p* ≤ 0.01, **** *p* ≤ 0.0001 relative to the control (PON1; rePON1 without bioactive lipid). Statistical analysis was via one-way ANOVA and Graphpad prism 9 software. See Figure 1 for compounds corresponding to each number.

The effect of the synthesized lyso-DGTS derivatives and analogs on rePON1 arylesterase activity was also examined. The arylesterase activity of rePON1 was significantly increased in a dose-dependent manner when incubated with the exogenous compounds **1** and **6** for 2 h at 37 °C, from 4 units without the lipid to 8 units at a concentration of 10 µg/mL and 10 units when incubated with 20 µg/mL. Compounds **2**, **3**, **4**, and **5** had no effect on the arylesterase activity of rePON1 compared to the control activity (Figure 3A).

Only two purchased endogenous lipids—lyso-PC (**7**) and lyso-PAF (**8**)—increased rePON1 arylesterase activity in a dose-dependent manner to about 10 units at a concentration of 10 µg/mL and to 12 units at a concentration of 20 µg/mL. The other endogenous lipids—lyso-PS (**10**), lyso-PA (**9**), and lyso-SM (**11**)—did not affect PON1 arylesterase activity (Figure 3B).

### 3.4. Interaction of Lyso-DGTS Derivatives and Endogenous Analogs with rePON1, Determined via the Trp Fluorescence-Quenching Method

**Figure 4:**
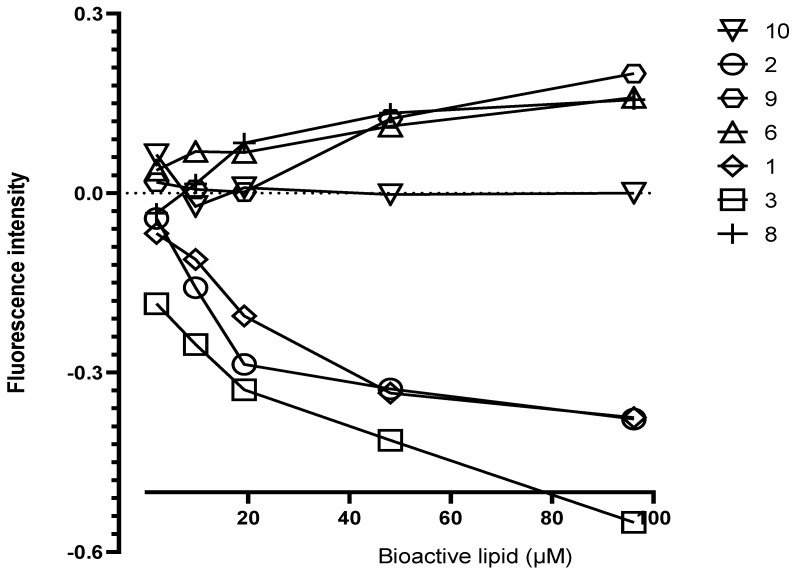
Effect of bioactive lipids on the Trp fluorescence of rePON1. rePON1 (50 µg/mL) was incubated with different bioactive lipids at various concentrations (0, 1, 5, 10, 25, 50, 75, or 100 µg/mL) in Tris buffer (pH 8.4). The fluorescence spectra were measured at an excitation of 290 nm and emission in the 320–340 nm range. Change in fluorescence was calculated as described in Experimental Procedures. Each experiment was repeated separately at least three times. See Figure 1 for compounds corresponding to each number.

Possible interactions between the bioactive lipids and rePON1 were examined by measuring the effect of the lipids on the fluorescence spectrum of Trp residues in rePON1. As demonstrated in Figure 4, three lipids—lyso-PA (**9**), lyso-PAF (**8**), and compound **6**—enhanced the Trp fluorescence intensity of rePON1 in a concentration-dependent manner up to 20 μM. However, three different lipids—the natural lyso-DGTS (**1**), compound **2**, and compound **3**—quenched the Trp fluorescence of rePON1 in a concentration-dependent manner. The other lipids, including lyso-PS (**10**), did not affect the Trp fluorescence of rePON1 (Figure 4).

### 3.5. Interaction between rePON1 and Lyso-DGTS Derivatives and Endogenous Analogs Using Molecular Modeling Calculation

**Figure 5:**
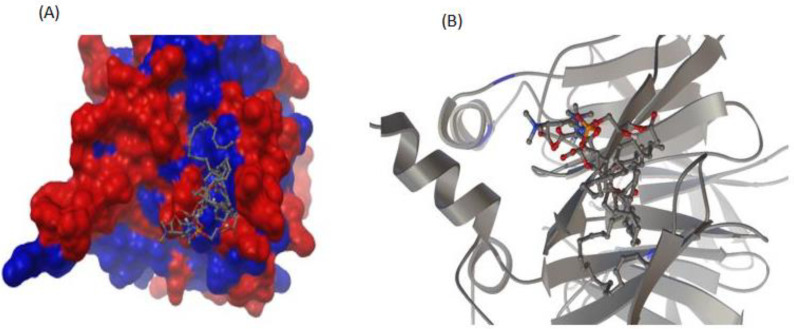
Some of the lipids in the rePON1 groove are orientated nearest α-helix H2. (**A**) The molecular surface of PON1 shows hydrophilic (red) and hydrophobic (blue) zones docked with the lipid. (**B**) Location of the lipid within the rePON1 groove showing interactions with the rePON1 groove using the secondary and tertiary structure of rePON1.

**Figure 6:**
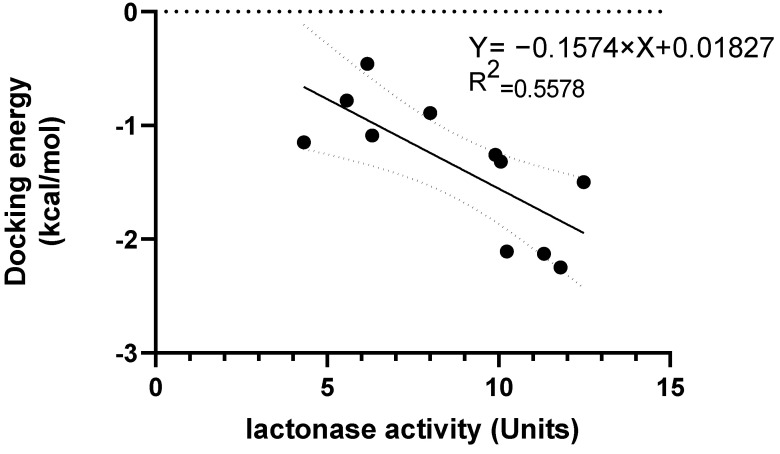
Correlation between the binding energy calculated from the AutoDock Tools program (Y-axis) and the rate at which PON1 catalyzes lactone hydrolysis (X-axis). The anticipated inverse correlation was obtained, with *R*^2^ = 0.5578 (*p* < 0.05).

PON1’s crystal structure was adjusted, and the lipids were constructed and docked on the enzyme’s crystallographic structure as described in Experimental Procedures. All of the bioactive lipids entered the same PON1 groove nearest the α-helix (H2), with very similar orientation, as shown in Figure 5. The fatty acid substructure of the lipid was oriented to the hydrophobic part of the groove (blue) and the polar substructure was oriented to the hydrophilic part (red). The inverse correlation (*R*^2^ = 0.5578, *p* < 0.05) between the lactonase activity of rePON1 (X-axis) and the theoretical docking energy of the interactions between rePON1 and the lipids obtained from the docking calculations (Y-axis) are shown in Figure 6 (lower docking energy represents stronger enzyme–lipid interaction).

### 3.6. Effect of rePON1–Lyso-DGTS Derivative and Analog Complexes on Cu^2+^-Induced LDL Oxidation

**Figure 7:**
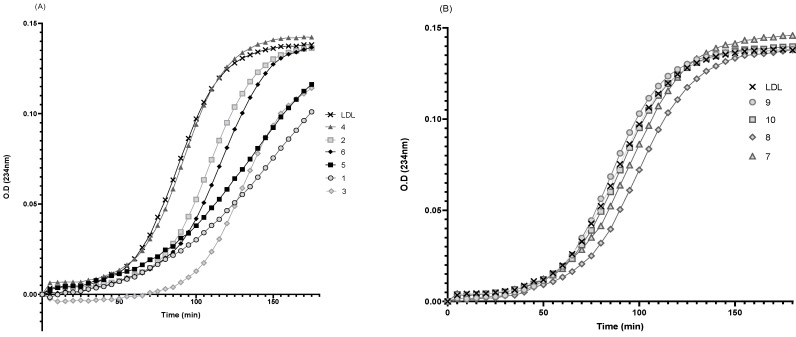
Effect of bioactive lipids on the oxidation of LDL induced by Cu^2+^. (**A**) The synthesized exogenous lipids 1–6 and (**B**) The purchased endogenous lipids 7–10. Conjugated diene formation during copper-mediated LDL oxidation was monitored for 180 min for changes in absorbance at 234 nm. Each experiment was repeated separately three times. Results are presented as mean ± SD. See Figure 1 for compounds corresponding to each number.

**Figure 8:**
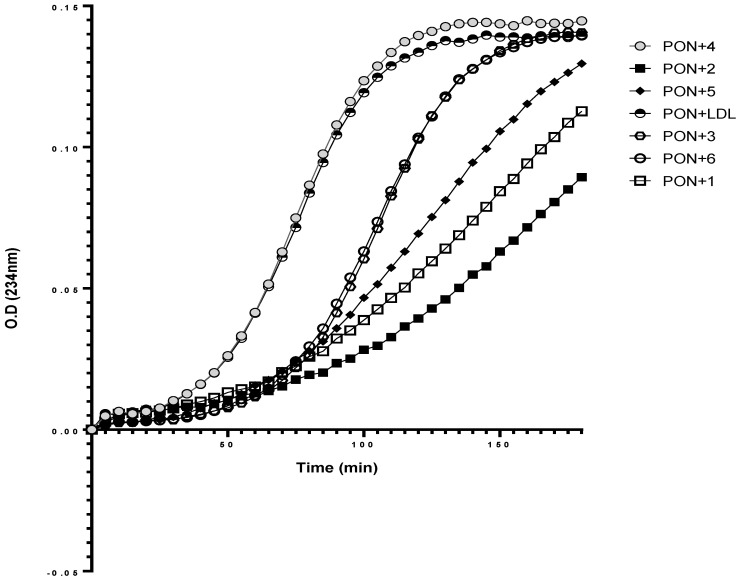
Effect of rePON1 with the exogenous bioactive lipids on the oxidation of LDL induced by Cu^2+^. Conjugated diene formation during copper-mediated LDL oxidation was monitored for 180 min for changes in absorbance at 234 nm. Each experiment was repeated separately at least three times. Results are presented as mean ± SD. See Figure 1 for compounds corresponding to each number.

The ability of the bioactive lipids, with or without rePON1, to prevent LDL oxidation induced by copper ions was examined. LDL oxidation is a radical reaction characterized by two parameters, lag time and propagation phase, which were calculated using nonlinear regression with the Gompertz growth equation. A representative kinetic analysis of LDL oxidation induced by the copper ions is shown in Figure 7 and Figure 8, and the oxidation parameters are summarized in Table 1 and Table 2. The LDL oxidation lag time increased from 24.2 min to 96.8, 99.4, 35.37, 29.37, and 36.25 min after incubation with lyso-DGTS (**1**) and the synthesized compounds **5**, **6**, **2**, and **4**, respectively. In addition, LDL oxidation was inhibited by 75%, 75%, 31.5%, 17.5%, and 33.16% after incubation with lyso-DGTS (**1**) and the synthesized compounds **5**, **6**, **2**, and **4,** respectively (Table 1). Compound **4** and the endogenous compounds **7** to **11** had no protective effect on LDL oxidation induced by copper ions (Figure 7B).

RePON1 was incubated with the exogenous lipids lyso-DGTS (**1**), **2**, **3**, **5**, and **6**, and its protective effect on LDL oxidation was also examined (Figure 8). As shown in Table 2, the complex of rePON1 with lyso-DGTS (**1)** inhibited LDL oxidation by 79%, and compounds **5**, **6**, **2**, and **3** inhibited LDL oxidation by 64%, 31%, 87%, and 16%, respectively. Compound **4** did not inhibit LDL oxidation. In addition, LDL oxidation lag time increased from 24.08 to 118, 67.87, 29.65, 199, and 28.73 min after incubation with rePON1 and lyso-DGTS (**1**) or the synthesized compounds **5**, **6**, **2**, and **4,** respectively.

## 4. Discussion

PON1 is a calcium-dependent HDL-bound enzyme that catalyzes the hydrolysis of multiple compounds, such as paraoxon (paraoxonase activity), arylesterase (arylesterase activity), and lactones (lactonase activity). Many of the antiatherogenic functions of HDL are attributed to PON1 [27]. PON1 enhances HDL cholesterol-mediated efflux from macrophages, protects LDL from oxidation by lowering lipid peroxide levels, inhibits ox-LDL uptake by macrophages thus inhibiting macrophage foam cell formation, and inhibits macrophage cholesterol biosynthesis [28]. PON1 antioxidant activity is inversely correlated to carotid intima-media thickness. The hydrolytic lactonase, arylesterase, and paraoxonase activities of PON1 are all inactivated under oxidative stress, and epidemiological evidence shows that low serum PON1 activity is associated with an increased risk of CVD [29]. Thus, enhancement of PON1 activity could be a useful approach to attenuating atherosclerosis development.

A previous study showed that phosphatidylcholine (PC) (16:0/18:2) specifically interacts with rePON1 and increases its lactonase and paraoxonase activities [28]. Recently, a betaine lipid derivative composed of EPA (C20:5) fatty acid connected to glyceryltrimethylhomoserine (C20:5 lyso-DGTS lipid) was isolated from an ethanol–water (70:30%) extract of *Nannochloropsis* sp. microalgae and was found to increase rePON1 lactonase and esterase activities [23]. In the present study, five new derivatives of lyso-DGTS were synthesized, and five additional endogenous lyso-lipids were purchased, and their effects on rePON1 lactonase and arylesterase activities were examined. The role of the glycerol moiety in lyso-DGTS activity was examined by connecting the fatty acids EPA, oleic acid, or palmitic acid directly to the N-methylated homoserine via an ester bond (compounds **2**, **3**, and **4**, respectively). In the two other synthesized compounds, EPA and oleic acid were connected directly to the N-trimethylhomoserine via an amide bond (**5** and **6**, respectively).

The natural compound lyso-DGTS (**1**) and the synthesized compound **6** strongly increased lactonase and esterase activities of rePON1, more than the other synthesized compounds. Compounds **4** and **5** increased the lactonase activity of rePON1, but to a lower level than compounds **1** and **6**. In contrast, compounds **2** and **3** did not affect the lactonase or esterase activities of rePON1 (Figure 2 and Figure 3). These results demonstrated that connecting the fatty acids oleic and EPA directly to the N-trimethylhomoserine through an ester bond without the glyceryl moiety attenuates its ability to enhance lactonase and esterase activities of rePON1; however, connecting it through an amide bond preserved these abilities, most probably due to the higher stability of the amide bond compared to its ester analogue. The lyso-DGTS amide derivative with oleic acid enhanced the lactonase and esterase activities of rePON1 more than that with EPA. It is worth noting that incubation of rePON1 with free fatty acids (EPA, oleic acid, or palmitic acid) or N-trimethylhomoserine did not have any effect on its lactonase and esterase activities (data not shown).

Five endogenous active lipids analogous to lyso-DGTS were purchased and their effect on the lactonase and esterase activities of rePON1 were examined. The structures of lyso-PC and lyso-PAF (platelet-activating factor) are similar to that of lyso-DGTS; however, their negative charge is located on the phosphate oxygen instead of the carboxylic acid moiety. Lyso-PC differs from lyso-PAF in that the ester group is replaced by an ether group in the latter. Lyso-PA (phosphatidic acid) and lyso-PS (phosphatidylserine) are negatively charged lipids, whereas lyso-SM (sphingomyelin) is a positively charged lipid under physiological conditions. Therefore, the effect of the lipid charge on rePON1 activities was also examined. Two lipids, lyso-PC and lyso-PAF (compounds **7** and **8**, respectively), enhanced lactonase and esterase activities of rePON1 by the same level, meaning that replacing the ester bond in the glycerol moiety with an ether bond did not affect the ability to enhance rePON1 activities. In contrast, the other lipids (compounds **9**, **10**, and **11**) did not affect rePON1 activities. These results demonstrate that phospholipids with a neutral charge under physiological conditions (compounds **7** and **8**) better enhance rePON1 activities than the negatively or positively charged phospholipids (compounds **9**, **10**, and **11**). Thus, lyso-DGTS lipid derivatives and analogs have specific interactions with PON1 with respect to enhancing its activity, independent of their emulsification properties.

PON1 is known to contain three helical regions; of these, two helices (H1 and H2) can associate with amphiphilic phospholipids. Three out of four Trp residues reside in or close to helical regions H2 and H3 of PON1 [28,30]. Therefore, a Trp fluorescence-quenching assay was carried out to determine possible interactions between rePON1 and the lyso-DGTS derivatives and analogs. The fluorescence-quenching behavior varied widely, from no effect (or even slight enhancement) to strong quenching, up to 100%. As demonstrated in Figure 4, lyso-PA (**9**), lyso-PAF (**8**), and compound **6** enhanced the Trp fluorescence intensity of rePON1 in a concentration-dependent manner, suggesting that some Trp residue-containing regions of rePON1 might be exposed to a relatively low level of these compounds. However, three different lipids, the natural lyso-DGTS (**1**), compound **2**, and compound **3**, quenched the Trp fluorescence of rePON1 in a concentration-dependent manner, suggesting a specific interaction between PON1 and these lipids. Lyso-PS (**10**) did not have any quenching effect. There was no correlation between the tested activities of rePON1 and the change in Trp fluorescence.

It was previously found that a specific PC interacts with PON1 near α-helix H2 [28]. The results obtained in the present study from the molecular docking analysis also showed that the examined lipids interact near the H2 site. There was a significant correlation between the lactonase activity of rePON1 and the binding energy (ΔG) obtained from molecular docking. H1 at the N-terminus of PON1 mediates its anchoring to HDL, and together, H2 and H1 form a hydrophobic patch that binds to the HDL surface. In addition to its role in HDL binding, H2 comprises part of the active site wall of PON1. The interaction site of the lipids with PON1 was near the α-helix H2. It is possible that this binding of the lipids to the allosteric site of PON1, near H2, might change its active-site conformation and enhance its affinity to the substrates, thereby elevating its activity.

Oxidation of LDL lipids and apolipoprotein B100 results in LDL in a proatherogenic process [31]. A clinical study suggested that a high ox-LDL concentration and a short LDL oxidation lag time might be independent risk factors for CAD; serum ox-LDL concentration was significantly elevated in CAD patients and the lag time of LDL oxidation was significantly shorter compared to controls [32]. We examined the ability of the bioactive lipids and their complexes with rePON1 to prevent LDL oxidation induced by copper ions. Two parameters were measured: the ability of lipids and the rePON1–lipid complex to inhibit LDL oxidation—represented by the lag time required for initiation of LDL oxidation and the reduction in lipoprotein-associated peroxides formed during 3 h of incubation—represented by the slope of the linear increase of the curve. As shown in Figure 7B, the endogenous lipids did not have any additional protective effect on Cu^2+^-induced LDL oxidation. Thus, lipids **7**, **8**, **9** and **10**, with fatty acids based on oleic acid, did not show any effect on antioxidant capacity. On the other hand, when the bioactive lipid was based on EPA (e.g., **1** and **5**), it increased the lag time by up to 95 min, and LDL oxidation was inhibited by about 75%, with or without incubation with rePON1.

EPA exhibited the most favorable molecular structure; the multiplicity of double bonds enhanced its ability to prevent LDL oxidation, as expected, and their absence had no effect on LDL oxidation (such as lipids **4** and **11**). A previous study showed that EPA has the optimal chain length and degree of unsaturation to inhibit oxidation of small dense LDL and membrane cholesterol domains [33].

## 5. Conclusions

In conclusion, the current study showed that the interaction of lyso-DGTS lipid and some of its derivatives with PON1 increases the latter’s activity, improves its lactonase and arylesterase activities, and prevents oxidation of LDL induced by copper ion. A clear understanding of the mechanism underlying the lipids’ role as antiatherogenic compounds, via the influence of their binding on the structural and biological activities of proteins (PON1) and lipoproteins, is key to the evaluation of these potent biomolecules’ contribution to human health and is a topic of active investigation in our laboratory.

## Figures and Tables

**Table 1:** Effect of the synthesized lipids on lag time, rate constant of the reaction propagation (K), and % inhibition of LDL oxidation. See Figure 1 for compounds corresponding to each number.

Compound	Lag Time	K	% Inhibition
LDL	24.22	0.04128	0
**1**	96.8	0.0103	75.04
**5**	99.4	0.01006	75.62
**6**	35.37	0.02827	31.51
**2**	29.37	0.03405	17.51
**4**	25.56	0.03913	5.20
**3**	80.50	0.02759	33.16

**Table 2:** Effect of rePON1 in a complex with the synthesized lipids on lag time, rate constant of the oxidation propagation (K), and % inhibition of LDL oxidation. See Figure 1 for compounds corresponding to each number.

Compound	Lag Time	K	% Inhibition
LDL	24.08	0.04152	0
**1**	118	0.00844	79.67
**5**	67.87	0.01473	64.52
**6**	29.65	0.03373	18.76
**2**	199	0.005013	87.9
**4**	23.73	0.04215	0
**3**	90.30	0.03481	16.16

## Data Availability

The data presented in this study are available in the article.
